# Seeing without Seeing? Degraded Conscious Vision in a Blindsight Patient

**DOI:** 10.1371/journal.pone.0003028

**Published:** 2008-08-21

**Authors:** Morten Overgaard, Katrin Fehl, Kim Mouridsen, Bo Bergholt, Axel Cleeremans

**Affiliations:** 1 CNRU, Hammel Neurorehabilitation and Research Center, Aarhus University Hospital, Hammel, Denmark; 2 Department of Psychology, Phillips-Universität Marburg, Marburg, Germany; 3 Center for Functionally Integrative Neuroscience, Aarhus University Hospital, Aarhus, Denmark; 4 Department of Neurosurgery, Aarhus University Hospital, Aarhus, Denmark; 5 Cognitive Science Research Unit, Université Libre de Bruxelles (U.L.B.), Bruxelles, Belgium; University of Southern California, United States of America

## Abstract

Blindsight patients, whose primary visual cortex is lesioned, exhibit preserved ability to discriminate visual stimuli presented in their “blind” field, yet report no visual awareness hereof. Blindsight is generally studied in experimental investigations of single patients, as very few patients have been given this “diagnosis”. In our single case study of patient GR, we ask whether blindsight is best described as unconscious vision, or rather as conscious, yet severely degraded vision. In experiment 1 and 2, we successfully replicate the typical findings of previous studies on blindsight. The third experiment, however, suggests that GR's ability to discriminate amongst visual stimuli does not reflect unconscious vision, but rather degraded, yet conscious vision. As our finding results from using a method for obtaining subjective reports that has not previously used in blindsight studies (but validated in studies of healthy subjects and other patients with brain injury), our results call for a reconsideration of blindsight, and, arguably also of many previous studies of unconscious perception in healthy subjects.

## Introduction

Blindsight patients, whose primary visual cortex is severely lesioned, exhibit preserved ability to discriminate visual stimuli presented in their blind field, yet report no visual awareness [1]. This finding has been widely interpreted as indicating that vision can occur unconsciously [2], and, for this reason, it is considered one of the most interesting sources of data in the study of human consciousness. A few studies, contrary to the standard interpretation of blindsight, have instead suggested a correlation between discrimination ability and conscious awareness, just as in healthy participants. Thus, both Zeki & Ffytche [3] and Stoerig & Barth [4] have reported findings of “weak visual experiences” in blindsight. Larry Weiskrantz and colleagues found that blindsight patient DB described experiencing “feelings” when presented with visual stimuli in his blind field [5]. Weiskrantz suggested that such blindsight patients should be distinguished from those who fail to report any conscious experience whatsoever (“Type 2” vs “Type 1” patients).

Here, we ask whether blindsight is best described as unconscious vision, or rather as conscious, yet severely degraded vision. Addressing this question requires using a sufficiently sensitive method to collect subjective reports. Most previous studies have used a binary report methodology (“Did you see the stimulus or not?”) [6], sometimes augmented by further probing about the patient's confidence in his report [7], [8]. In all such cases however, asking participants to express a binary judgment about their perceptual experiences may fail to detect weak conscious knowledge, and hence underestimate the extent to which participants are aware of such knowledge. Lau and Passingham [9] have recently presented the similar suggestion that blindsight patients may have conscious vision, but set their threshold for reporting awareness too high.

The patient involved in the experiment below is a 31-year old woman (GR) who had been experiencing fluctuating attacks of headaches during a period of 2–3 months. She suddenly developed strong headache, blindness in the right part of her visual field, and she dropped to a Glasgow Coma Score (GCS) level of 12. Cerebral CT scanning showed a hemorrhage in the left occipital lobe (anterior part), surrounding subarachnoid space, and into the left lateral ventricle. There was a moderate hydrocephalus.

The following day, GCS decreased to 7, and a new CT scanning showed increased hydrocephalus. A drain was placed in the right lateral ventricle to drain CSF. Angiography showed an arteriovenous malformation. The malformation was treated with endovascular embolization. After surgery, she gradually woke up, but was disoriented and showed decreased short term memory and right sided hemianopia.

A control CT scanning 1.5 weeks later showed that the bleeding was resorbed, revealing loss of tissue in the left occipital cortex.

GR left intensive care and started rehabilitation at Hammel Neurorehabilitation and Research Center. Here, she slowly recovered from all physical and cognitive dysfunction, except the right sided hemianopia. An opthamological examination concluded that she had no dysfunction or injuries to the eyes, and it was concluded she was “cortically blind” in the upper right quadrant. The experiments were conducted after GR had been in rehabilitation for about 3 weeks, and her neuropsychological performance seemed stable. In a telephone interview a year after the study, her condition was not changed from at the time of the experiments.

## Results and Discussion

To obtain more exact subjective reports, we developed a novel method to assess awareness in healthy participants, — the Perceptual Awareness Scale (PAS) [10]. To develop PAS, we asked how healthy participants spontaneously scale the clarity of their perceptual experiences when presented with visual figures (triangles, circles and squares) displayed for a random duration ranging between 16 and 192 ms. All subjects intuitively chose a multiple point scale and found it to represent their visual experience better. Interestingly, the subjects generally agreed on the following labelling of four scale points: (CI) “clear image”, (ACI) “almost clear image” (meaning “I think I know what was shown”), (WG) “weak glimpse” (meaning “something was there but I had no idea what it was”), and (NS) “not seen” [10]. In subsequent experiments [11], the PAS scale has been adapted to be used by other participants, who again interacted with the experimenter about the meaning of the scale points. All participants in these further studies found the scale to accurately reflect their visual experiences. Strong correlations to accuracy and reaction time were also found for each participant: The more clearly the subjects reported to see the stimulus, the faster they responded what it was and their answer tended to be correct.

Here, for the first time, PAS is applied to assess awareness in a blindsight patient, GR, who reports no visual experience in the upper right quadrant of her visual field after damage to the left part of her visual cortex. The scale shares basic similarities with a scale created by Zeki and Ffytche^4^ in a study of GY, a patient who was hemianopic after a lesion to V1. GY claimed to have vague feelings of stimuli presented to his blind field. Zeki and Ffytche suggested that lesions to V1 may lead to an uncoupling of visual discrimination and awareness. Presenting GY to moving stimuli, authors demonstrate that activity in V5 is more intense in gnosanopsia (awareness without discrimination) than in agnosopsia (discrimination without awareness). The scale captured the essence of GY's “residual awareness” in his blind field, which he described as “a feeling of something happening”, although the scale points were not based on empirical grounds in the same way as PAS. Essential to the finding is that these kinds of experience cannot fit into a dichotomic division between clearly conscious and unconscious perception.

**Figure 1:**
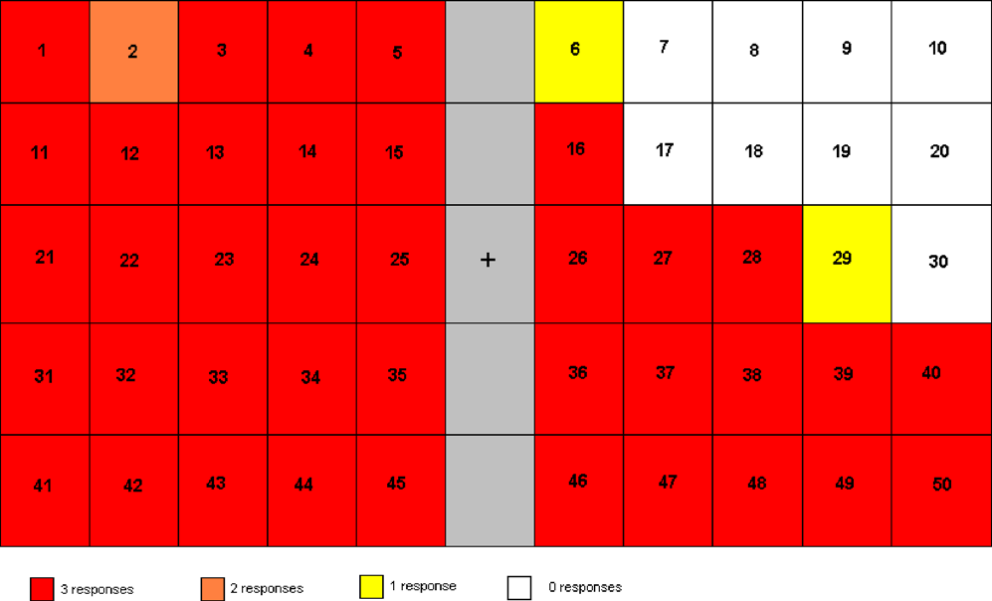
Experiment 1 reveals the size and location of GR's blind field. This figure illustrates how the screen was divided into areas, where stimuli were presented. For each numbered area, 3 stimuli were presented in random order during the experiment. The colour code illustrates how many times she responded to stimuli flashed in the relevant part of the screen

In a first experiment aimed at documenting the extent of GR's deficit, the patient was asked to indicate whether she had perceived a letter presented on a computer screen. As expected from GR's clinical examination, she missed all presentations in the upper right quadrant (figure 1). At the periphery of the blind area, there are two stimulus locations with one instead of zero hits out of three presentations. This can be interpreted as resulting either from saccadic eye movements, or as indicating a “border area” where vision is partially intact. As her task was simply to report detection “of something”, her lack of responsiveness in the upper right quadrant clearly indicated no normally functioning conscious vision in the area.

In a second experiment, we aimed at exploring the relationship between GR's discrimination performance and her visual awareness, using standard “objective” methods for assessing awareness. To do so, GR was asked (1) to guess which figure was presented to her, and (2) to report whether she saw something or not, using binary report.

The results from experiment 1 were used to select the results that were to be compared in the following two experiments. On this basis, 11 locations with 0 or 1 response were selected. The stimuli presented in the upper right quadrant (33 during experiment 2, and the same amount for experiment 3) were compared to the stimuli presented in the upper left quadrant (a directly comparable “healthy part” of her visual field with the same amount of stimuli). As shown in Table 1a, there are, as expected, many more stimuli reported to be “unseen” rather than “seen” in the damaged part of GR's visual field (26 “unseen” items vs 7 “seen”). Amongst the 7 stimuli reported “seen”, 6 were correctly identified. Of the 26 stimuli that were reported “unseen”, 12 were nevertheless correctly identified. There is thus no relationship between accuracy and awareness (p = 0.15 using Pearson's Chi-squared test with Yates' continuity correction, p = 0.1 using Fisher's Exact Test for count data) for the injured visual area under these conditions. This result contrasts with those obtained for the intact upper left quadrant of the visual field: 27 stimuli were reported to be “seen”, vs. 6 reported to be “unseen”. All 27 seen stimuli were correctly identified. Of the 6 stimuli that were reported “unseen”, 2 were correctly identified. There is thus a significant relationship here between accuracy and awareness (p = 0.0001, using Pearson's Chi-square with Yates' continuity correction, p = 0.0003 using Fisher's Exact Test for count data): For the intact left quadrant of GR's visual field — but not for the damaged right quadrant — reports of perceptual consciousness are predictive of visual function, as is the case in healthy subjects^13^. There was no significant effect of stimulus duration. According to the definition of blindsight, — “visual capacity in a field defect in the absence of acknowledged awareness”^ 1^, GR exhibits blindsight in the damaged part of her visual field.

**Table 1:** a–c: The number of correct and incorrect reports using dichotomous reports (1a) or using PAS (1b).

	Intact field		Injured field	
	Correct	Incorrect	Correct	Incorrect
Seen	27	0	6	1
Not seen	2	4	12	14

	Intact field		Injured field	
	Correct	Incorrect	Correct	Incorrect
CI	21	0	7	0
ACI	8	1	8	3
WG	1	2	3	9
NS	0	0	0	3

	Intact field		Injured field	
	Correct	Incorrect	Correct	Incorrect
CI-ACI	29	1	15	3
WG-NS	1	2	3	12

The difference is illustrated in a “dichotomizing” of PAS (1c).

The third experiment was identical to the second, with the crucial difference that reports of awareness were now collected using PAS instead of binary report. Following PAS methodology, GR went through many trial sessions and discussed with the experimenters how to best characterize her experiences. She indicated that the PAS scale points accurately reflected her experiences in the framework of this experiment, and that PAS offered a better, more precise match to her perceptions than binary report.

**Figure 2:**
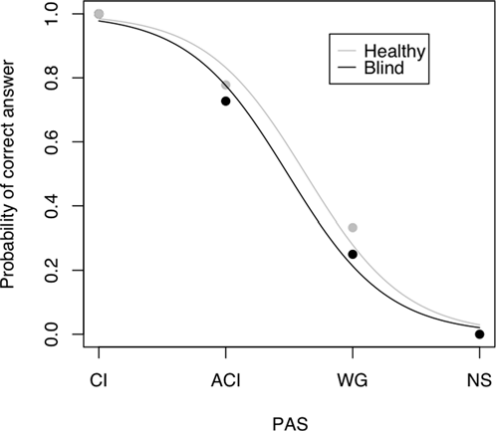
A regression analysis illustrates the relationship between correctness and PAS. The analysis reveals that the relationship between accuracy and awareness as assessed by PAS is the same in the intact and in the blind fields.

As shown in table 1b, GR never reported “not seeing anything” in the healthy part of her visual field. These results confirm the expected strong relationship between experiential clarity and accuracy as illustrated in figure 2. Strikingly however, and in contrast to the results obtained using binary report, we observed the same positive relationship between experiential clarity and accuracy for items presented in the patient's blind field. A chi^2^ test showed 14.2, p<0.001 for the intact visual field and 15.1, p<0.002 for the “blind” field.

This in turn suggests (1) that awareness, when properly assessed, is predictive of accuracy in the case of blindsight, and (2) that PAS is a more sensitive measure of conscious awareness than dichotomous reports.

**Figure 3:**
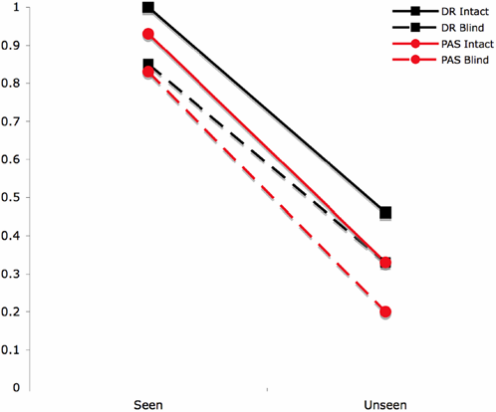
Probability of correctly identifying the stimulus given a “seen” vs. “unseen” report (Dichotomous Report) or given a “Clear Experience/Almost Clear Experience” vs. a “Weak Glimpse/Not seen” report (PAS report), plotted separately for the Intact and Blind fields".

To further illustrate the relationship between PAS and binary report, we split the PAS data in different ways. We note that the pattern of correct and incorrect reports obtained by pitting PAS “clear image” reports against the other three categories is almost identical to that obtained when GR was asked to gave binary reports. This suggests that GR is setting her threshold for reporting awareness too high under binary report conditions. If, instead, the cut-off for awareness is placed between scale points “almost clear image” and “weak glimpse” (Table 1c) we would conclude that GR has a high degree of awareness of stimuli presented in the injured part of her visual field, since there is a correlation between awareness and accuracy (p = 0.0004, Fisher's exact test). An illustration of turning the PAS reports into a binary measure is also shown in figure 3.

To conclude, with GR, we have demonstrated blindsight using a visual discrimination task and a standard measure of visual awareness (Experiment 2). As such, the experiment replicates the findings of numerous previous studies. Experiment 3, however, suggests that GR's ability to discriminate amongst visual stimuli does not reflect unconscious vision, but rather degraded, yet conscious vision. Because this result critically depends on using a sensitive method to assess awareness, our study therefore calls for a reconsideration of the methodologies and results obtained in previous studies not only of blindsight, but also of normal vision.

## Materials and Methods

In experiment 1, on each trial, GR was first asked to fixate a white central cross appearing on a black background. One of three letters (chosen randomly among A, B, or C) was then presented at one of 50 equidistant locations spanning the entire visual field, for a duration of 60 ms, 500 ms, or 1000 ms. All stimuli were 1.4*1.4 cm on the screen. Each location corresponded to one of the fields in figure 1. Stimuli were presented with the different durations randomly. The patient then had 3000 ms to indicate having seen the stimulus by pressing a key on a computer keyboard. The next trial was initiated immediately thereafter, but to avoid temporal predictability of the next stimulus, the fixation cross remained on screen for a random duration ranging from 3 to 6 sec (in increments of 1 sec). The entire experiment involved three blocks of 50 trials separated from each other by 10 min breaks, for a total of 150 trials (50 locations×3 durations). Out of these 50 locations, 11 were selected for more detailed statistical analysis based on the results from experiment 1, as described above. Locations and durations were fully randomized.

In experiment 2 and 3, GR performed a 3-choice discrimination task presented with one of three possible white geometrical figures which were displayed in random order: a triangle, a circle or a square. The change from letters to geometrical figures as stimuli, we believed, would help the patient remember that these tasks differed from the first. As for Experiment 1, each trial was initiated by the appearance of a white fixation cross displayed on a black background for a random duration ranging between 3 and 6 sec. This was followed by the stimulus, randomly presented for 60, 500, or 1000 ms. Following every stimulus presentation, a mask was presented for 1000 ms. Stimuli were presented once per duration at every stimulus location (identical to experiment 1), i.e. a total of 150 trials. After every 50 trials, there was a break for 10 minutes.

After the mask, in experiment 2, GR was presented with a still screen picture asking her to report whether she had consciously seen the stimulus or not, pressing one of two buttons. Upon her report, another still screen picture was presented, asking her to identify which of the three stimuli that was presented, pressing one of three buttons. In experiment 3, the first still screen picture was identical to the one in experiment 2. The second still picture showed the PAS categories together with the four buttons used.

The experiments were approved by the local ethical committee, The Central Denmark Region Committee on Biomedical Research Ethics. GR's written and verbal consent were obtained prior to the experiments. The patient's written and verbal consent were obtained prior to the experiments. The patient has given written informed consent to the publication of this article.
